# The Medical Congress at Calcutta

**Published:** 1895-01-19

**Authors:** Ernest Hart


### THE MEDICAL CONGRESS AT CALCUTTA.

(COMMUNICATED BY MRS. ERNEST HART.)

The first meeting of an Indian Medical Congress is an event of great importance to the medical profession in India. The Congress is held under the presidency of the Viceroy, and was opened in person by His Excellency Lord Elgin on Monday, December 24th, 1894. Surgeon-Colonel Harvey, in his presidential address, said that from a very early time native doctors in India were trained at the local dispensaries, beginning as apprentices or even as hospital coolies, picking up what they could at the bedside, aided by informal teaching from the civil surgeon, and a great step forward was made when in 1822 a vernacular medical school was started in Calcutta. By sanitation the annual death-rate of 69 per 1,000 has been reduced to 15 per 1,000. During the last 30 years medical education and the provision of the means of giving medical relief have made enormous strides. There are now 2,025 hospitals and dispensaries in India. Yet Dr. Harvey deplored the need for more hospitals and more dispensaries, and speaking with hearty sympathy of the work and aim of the Dufferin Fund, he said he trusted to see in every important town in India a Dufferin Hospital side by side with its hospital for males. In sanitation the progress has also been most marked. Many cities are now supplied with pure pipe water. Village sanitation Acts, though permissive, are being extended. Sanitary boards, on which is a sanitary engineer, are now established in most provinces. The obligations of Government to deal with preventable diseases are more fully recognised. But though so much had been done, still much more remains to be done. Dr. Harvey speaking as an old teacher and examiner, said that, in spite of all drawbacks, the medical education given in the medical colleges which teach in English was marvellously good. He urged the extension to India of some such Act as the Medical Acts of England, and the formation of a body like the General Medical Council. Dr. Harvey pointed out that an anna, a trifle more than three farthings, per head of the population paid to a special sanitary fund would produce in round numbers fourteen millions of rupees, and would supply not only a health officer for every district in India, but would leave a very large balance available for sanitary works. After dwelling upon other topics, the President concluded his address with a note of encouragement to all. A number of papers were read, amongst the most interesting being Dr. Crombie's paper on "Malarial and Non-malarial Remittent Fevers," and that of Professor Haffkine on "Inoculations for Cholera." The weather is perfect, like a brilliant June, and the Indian Medical Congress is being inaugurated under auspicious circumstances.

